# Glottic and skull indices in canine brachycephalic airway obstructive syndrome

**DOI:** 10.1186/1746-6148-10-12

**Published:** 2014-01-11

**Authors:** Roberta Caccamo, Paolo Buracco, Giuseppe La Rosa, Matteo Cantatore, Stefano Romussi

**Affiliations:** 1 Department for Veterinary Science, Faculty of Veterinary Medicine, University of Turin, Grugliasco, Italy; 2 Veterinary Practitioner, Turin, Italy; 3 Hospital for Small Animals, Royal (Dick) School of Veterinary Studies, Easter Bush Veterinary Centre, Roslin, Midlothian EH25 9RG, UK; 4 Department for Veterinary Sciences and Public Health, Faculty of Veterinary Medicine, University of Milan, Milan, Italy

**Keywords:** Canine brachycephalic airway obstructive syndrome, Skull measurements, Glottic measurements, Glottic index, Laryngeal collapse

## Abstract

**Background:**

Forty dogs presented for brachycephalic airway obstructive syndrome with laryngeal collapse not over 1st degree (saccule eversion) underwent glottis endoscopic and radiographic skull measurements before surgery. Fifteen Pugs, fifteen French and ten English Bulldogs were included. The goals were prospectively to compare three common brachycephalic breeds for anatomical differences regarding glottis and skull measurements, and to assess if any correlation between glottis and skull measurements was present. Linear measurements were used to obtain glottis and skull indices. Correlations between glottis and skull indices and glottic measurements were evaluated. Finally, glottis indices were compared among the three breeds.

**Results:**

No correlation was found for glottis and skull indices. The glottic index differed among the three breeds (smaller in Pugs and higher in English Bulldogs), ultimately representing a morphologic indicator of the different larynx shape in the three breeds (more rounded in English Bulldogs, more elliptical in Pugs and in-between in French Bulldogs).

**Conclusions:**

The lack of correlation between skull/glottic indices does not support skull morphology as predictor of glottic morphology. As Pugs had the lowest glottic index, it may be speculated that Pugs’ original narrow glottic width may predispose to further progressive respiratory deterioration more easily than in the other two breeds.

## Background

Brachycephalic airway obstruction syndrome (BAOS) is a well-known combination of several upper airway abnormalities [1-3]. These abnormalities may be defined as primary (stenotic nares, elongated and thick soft palate, excessive nasopharyngeal turbinates, and hypoplastic trachea) [4-6] or secondary (mainly laryngeal collapse) [7]. Redundant and hypertrophied pharyngeal folds, macroglossia, laryngeal edema, enlarged tonsils, and bronchial collapse may also be present [8-10]. Respiratory signs (snoring, coughing, stertor, stridor, dyspnea, tachypnea, exercise intolerance, and cyanosis/syncope) are usually progressive, and their severity depends on the degree of airway obstruction [3]. Gagging, retching, regurgitation and vomiting, along with other lower gastrointestinal signs (such as dilated stomach and flatulence caused by aerophagia) may be associated [11-13].

To our knowledge, there are no published studies that evaluate inter-breed variations of endoscopic larynx examination and, more specifically, of glottic anatomy in brachycephalic dogs. Furthermore, no study has investigated endoscopic glottic measurements in brachycephalic breeds, or assessed laryngeal and skull morphology as potential predictors for BAOS progression. As laryngeal collapse appears as one of the most threatening secondary effects of airway obstruction during the progression of BAOS, authors’ goal was to look for potential differences of the laryngeal shape among three common brachycephalic canine breeds presented for BAOS surgery. For this purpose only symptomatic dogs for BAOS with not over a first degree laryngeal collapse at endoscopy were chosen. The study was divided in two prospective phases. *Phase I*: laryngeal morphometric study comparing English and French bulldogs and Pugs. *Phase II*: evaluation of the correlation between endoscopic glottic and radiographic skull measurements. Besides, glottis indices were compared among the three breeds. Attention was also driven to ascertain if the larynx shape (mainly a narrow glottis opening) could be predicted based on the morphometric study of the skull.

## Methods

Dogs of three brachycephalic breeds (Pug, French Bulldog and English Bulldog) diagnosed with BAOS at the School of Veterinary Medicine of Milan and Turin between January 2006-December 2009 were consecutively enrolled in the study. Patients were excluded from the study if airway endoscopy identified the presence of laryngeal collapse more severe than grade I [7].

This prospective study complied with institutional guidelines for research on animals, and signed consent was obtained from all owners, and all procedures were part of standard diagnostic work-up and treatment.

Historical and physical examination data were collected for each animal. In all dogs, work-up prior to anaesthesia included: a complete blood count, an extensive biochemical serum profile and a cardiac ultrasonographic examination. In order to proceed with the laryngoscopic and tracheobronchoscopic exam (see later, Phase I), intramuscular premedication included 0.2 mg/kg methadone alone, or in combination with 10 μg/kg acepromazine. Lateral and dorso ventral radiographs of the chest were also taken at this time, also in order to check for tracheal hypoplasia, as established by the tracheal diameter/thoracic inlet ratio (TI) [8]. Pre-oxygenation was provided for 5 min prior to endoscopy with 100% oxygen (approximately 2 L/min) via a face-mask. General anaesthesia was induced with 2-4 mg/kg intravenous propofol; intravenous methylprednisolone sodium succinate (1 mg/kg) was given to control laryngeal edema; intravenous cefazolin (20 mg/kg) was also administered. Light plane of anesthesia (with spontaneous breathing and laryngeal function preserved) was maintained via propofol boluses, as required. Oxygen was delivered via a catheter alongside the endoscope. After completion of the endoscopic evaluation, dogs were intubated, and anesthesia was maintained with isoflurane in oxygen. Surgical correction of BAOS, performed by two different surgeons (SR and PB), consisted of vertical wedge nostril plasty and partial staphylectomy [14]. For soft palate resection the midpoint of the tonsils was used as the guide [15]. Laryngeal saccule resection was performed at the surgeons’ discretion. Prior to recovery from anesthesia, a dorso-ventral skull radiograph was taken.

### Phase I

Laryngoscopy was performed (SR and RC) with dogs in sternal recumbency, either using a 5.3-mm × 60-cm flexible videobronchoscope (Fujinon EB 250S, Fujinon Inc., Wayne, New Jersey) or a 5.0-mm X 55-cm flexible fiberbronchoscope (Olympus BF-P40, Olympus Medical Systems Europe GmbH, Hamburg, Germany). Images and movies of laryngeal motions were acquired using a video recording device (Sony GV-D1000E, Sony Corporation, Tokyo, Japan). Further images were obtained from movies using the computer software: Matrox Pc-Vrc Remote 2.0. The degree of laryngeal collapse was endoscopically assessed based on Leonard’s classification [7]. In particular, according to this classification, saccule eversion is considered as 1st degree laryngeal collapse. Progression of the disease leads to collapse of the cuneiform processes of arytenoids (2nd degree collapse) and can proceed to collapse of the corniculate processes of arytenoids (3rd degree collapse).

Glottic linear measurements, expressed in pixels, were taken of arytenoids at their most adducted position (end of expiration) using the UTHSCSA Image Tool 3.00 for Windows software. Measurements were: a) linear glottic height [dorso-ventral diameter (dorsal to ventral commissure)], b) linear glottic width [transversal diameter (distance between the vocal processes of the two arythenoid cartilages)], and c) glottic index as a result of glottic width × 100/glottic height. Glottic index was calculated according to skull index formula (see Phase II). To minimize bias, the arithmetic average of three consecutive measurements was calculated.

### Phase II

Digital dorso-ventral radiographs of the skull were taken (Agfa ADC SOLO CR System, Agfa HealthCare NV, Mortsel, Belgium). The head of the dog was kept parallel to the radiological table using a cushion under the chin. Images were analyzed with the UTHSCSA Image Tool 3.00 for Windows software. Linear measurements, expressed in pixels, were: a) skull length [inion-prosthion (central surface point on external occipital protuberance-anterior end of interincisive suture, between the roots of the upper central incisor teeth)], b) maximum zygomatic width [zygion-zygion (the most lateral point of the zygomatic arch)], and c) skull index (maximum zygomatic width × 100/skull length) [16,17]. To minimize bias, the arithmetic average of three different measurements was calculated.

### Statistical evaluation

Data were expressed as mean ± SD (standard deviation), median and range. Descriptive statistical analysis was used for age, body weight, and glottic and skull measurements. Data concerning everted saccules, hypoplastic trachea, and bronchial collapse were expressed as percentages. For all variables, the Shapiro-Wilk test of normality was applied. Correlation among age, body weight, and glottic and skull measurements was evaluated; Spearman’s rank correlation test and Pearson’s product moment correlation were applied to non-normally and normally distributed variables. The Wilcoxon rank sum test was used to compare age, body weight and glottic and skull measurements. A *P* value of <0.05 was considered statistically significant for all tests. To evaluate the repeatability of glottic measurements, the British Standards Institution repeatability coefficient was applied [18]. All statistical analyses were performed using the public domain program R 2.3.0 (R Development Core Team).

## Results

Forty dogs affected with BAOS were included in the study, subdivided as follows: 15 Pugs (37.5%), 15 French Bulldogs (FB, 37.5%), and 10 English Bulldogs (EB, 25%). The mean age was 2.64 ± 1.59 SD (median 2.5 years, and range 9 months to 6 years). In Pugs the mean age was 2.55 ± 1.2 SD (median 2.5 years, range 11 months to 5 years), in FB 2.85 ± 1.95 SD (median 2.5 years, range 9 months to 6 years), and in EB 2.44 ± 1.63 SD (median 2.5 years, range 9 months to 5 years). There was no significant age difference between the three breeds (*P* >0.05). Twenty-five of the dogs were male (62.5%), whereas fifteen were females (37.5%). The mean body weight was 14.02 ± 8.29 SD (median 10 kg, range 6–40 kg). In Pugs the mean body weight was 8.51 ± 1.34 SD (median 8.7 kg, range 6–11 kg), in FB 10.7 ± 2.17 SD (median 11 kg, range 7–12 kg) in FB, and in EB 26.6 ± 6.33 SD (median 24.5 kg, range 21–40 kg) in EB. Significant differences in body weight (Wilcoxon Rank Sum test) were found among Pug vs FB (*P* = 0.048), Pug vs EB (*P* = 0.002), and FB vs EB (*P* = 0.005).

Upon thoracic radiography, 6 dogs (15%) had tracheal hypoplasia (Pugs 0%; 1 FB, 16.7%; 5 EB, 83.3%). All dogs had both an elongated soft palate and stenotic nares; both conditions were surgically corrected. Twenty-seven dogs (67.5%) had everted saccules (10 Pugs, 37.0%; 9 FB, 33.3%; 8 EB, 29.6%) and 21 (77.8%) of these underwent laryngeal saccule resection (9 Pugs, 8 FB and 4 EB). Bronchoscopy also revealed that 4 dogs (10%) had bronchial collapse (2 Pugs, 50%; 0 FB, 0%; 2 EB, 50%).

### Phase I

**Figure 1:**
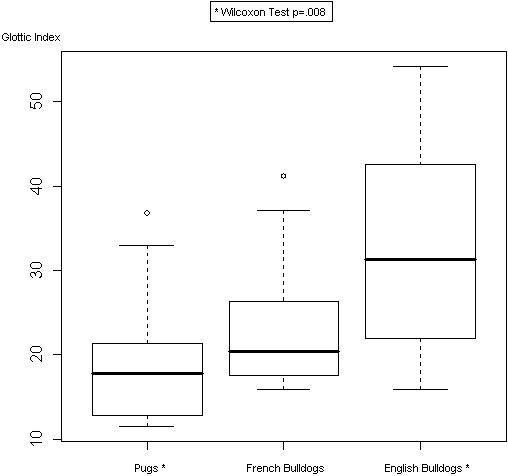
**Boxplot of the glottic index in each of the three breeds.** Pugs (Median 17.80, 1st Quartile 12.32, 3rd Quartile 21.79); FB (Median 20.45, 1st Quartile 17.29, 3rd Quartile 26.23); EB (Median 31.39, 1st Quartile 22.02, 3rd Quartile 41.88).

**Figure 2:**
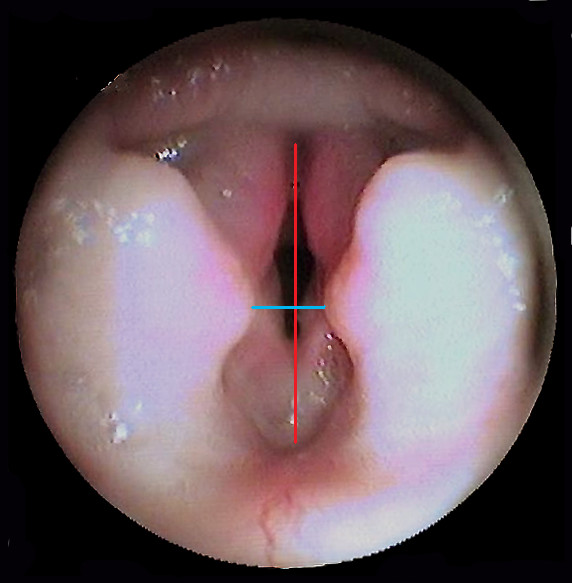
Endoscopic images of the larynx of a Pug, with linear measurements (transversal diameter blue color, longitudinal diameter red color).

**Figure 3:**
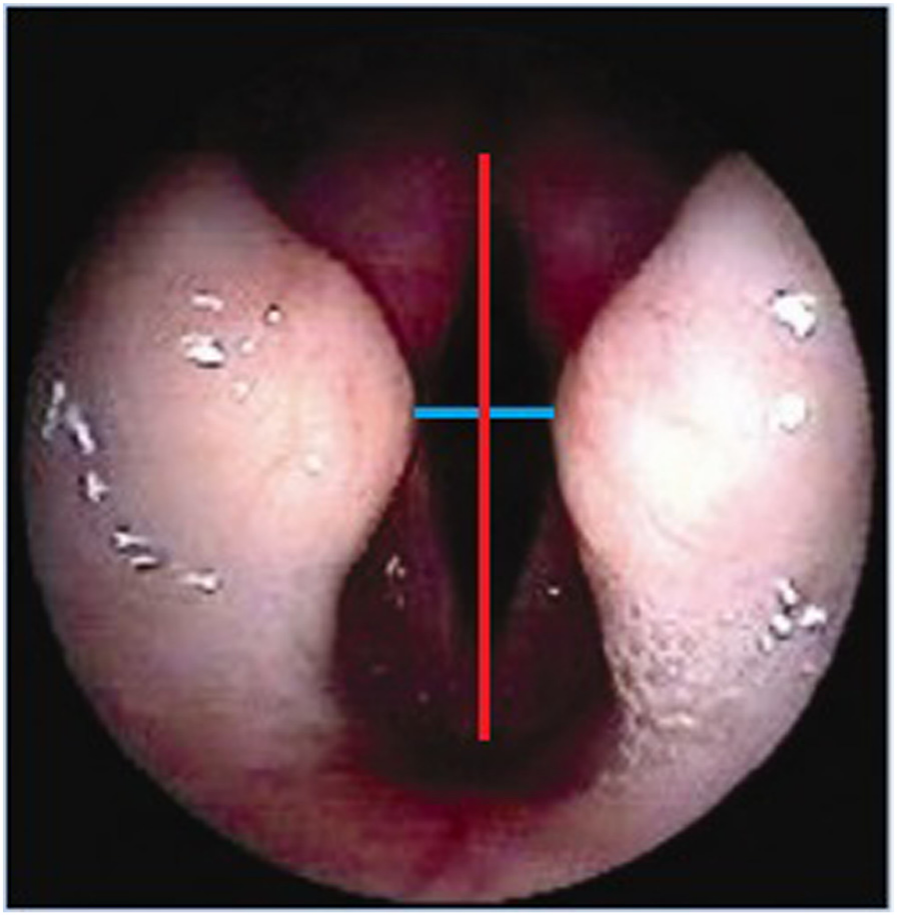
Endoscopic images of the larynx of an EB, with linear measurements (transversal diameter blue color, longitudinal diameter red color).

Laryngeal linear measurement and glottic index-related means, both expressed in pixels, are reported in Table 1. Analysis of direct measurements of the glottis did not show a significant difference among the three breeds. The average difference between repeated measurements of the glottis (*d*) was 2.75% (SD = 1.02%) for the first operator and 1.05% (SD = 0.6%) for the second one; the British Standards Institution repeatability coefficient (1.96 SD) was 1.99% and 1.18%, respectively. Glottic index showed an increasing trend, being smaller in Pugs and progressively bigger in French and English Bulldogs. In particular, a significant difference was clearly observed between Pugs and English Bulldogs (*P* = 0.008), with Pugs having the glottic index significantly smaller (i.e. narrower shape of the glottis) when compared to English Bulldogs (Figure 1); Figures 2 and 3 show laryngoscopic images in 2 dogs (1 Pug and 1 EB). Besides, no significant correlations between age and glottic index were found. Finally, the glottis index and body weight were not significantly correlated.

**Table 1:** Mean values obtained from glottic measurements

		**Glottic height**		**Glottic width**		**Glottic index**	
	**n**	**Mean ± SD**	**Median**	**Mean ± SD**	**Median**	**Mean ± SD**	**Median**
Total population	40	209.67 ± 46.41	223.12	48.70 ± 17.57	42.99	24.44 ± 10.81	20.71
Pugs	15	211.80 ± 28.16	223.12	39.97 ± 14.88	36.20	19.35 ± 8.06	17.80
FB	15	221.56 ±43.72	230.67	51.03 ± 16.61	45.30	23.58 ± 7.60	20.45
EB	10	186.31 ± 67.90	178.71	58.32 ± 18.04	53.07	33.35 ± 13.53	31.39

The only significant difference is between Pugs vs English Bulldogs (Wilcoxon Rank Sum test P = 0.008).

### Phase II

**Figure 4:**
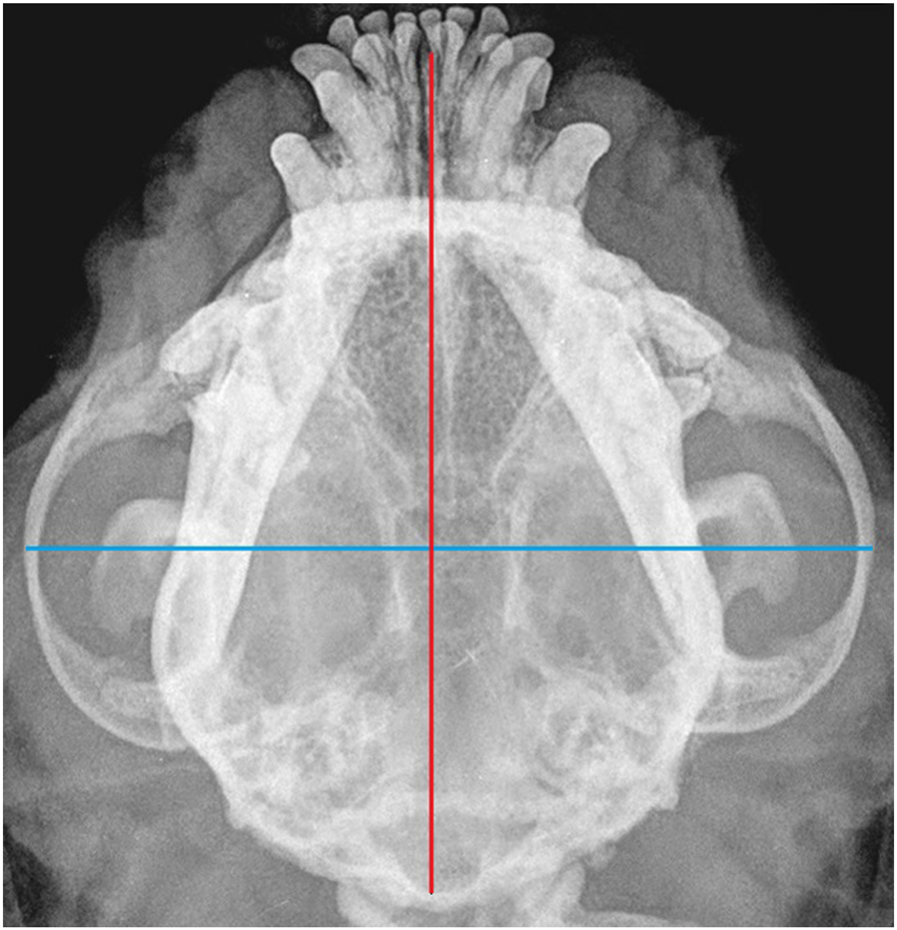
Radiograph of the skull of a Pug, with linear measurements (transversal diameter blue color, longitudinal diameter red color).

**Figure 5:**
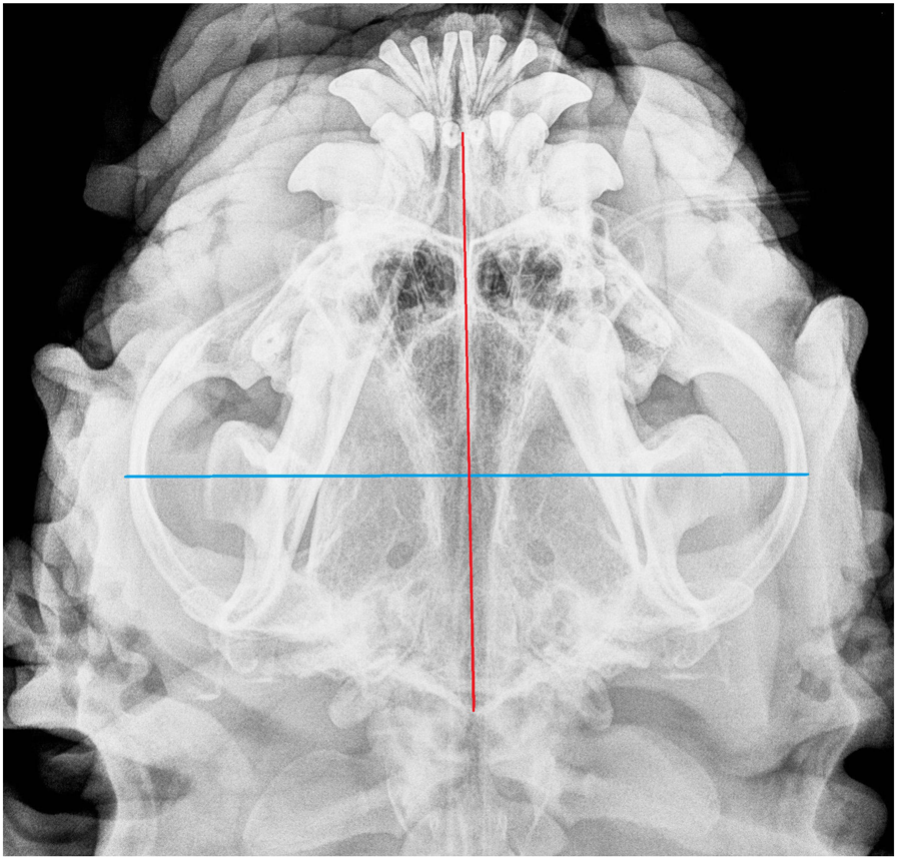
Radiograph of the skull of an EB, with linear measurements (transversal diameter blue color, longitudinal diameter red color).

Skull linear measurement and skull index-related means, both expressed in pixels, are reported in Table 2. No statistical correlation, calculated according to Pearson’s parametric correlation test and Spearman’s Rank sum test, between glottis and skull linear measurements and glottis and skull indices, were found, both in the entire population and among the three breeds. Comparison of the skull index among the three breeds (Wilcoxon Rank Sum test) did not show any significant difference. Figures 4 and 5 show skull radiographs in 2 dogs (1 Pug and 1 EB).

**Table 2:** Mean values obtained from skull measurements

		**Skull length**		**Maximum zygomatic width**		**Skull index**	
	**n**	**Mean ± SD**	**Median**	**Mean ± SD**	**Median**	**Mean ± SD**	**Median**
Total population	40	989.49 ± 220.32	917.08	1065.15 ± 239.29	1091.51	108.04 ± 11.99	108.55
Pugs	15	794.84 ± 68.82	793.5	803.94 ± 86.09	802.10	101.39 ± 9.33	101.83
FB	15	987.57 ± 130.88	936.36	1153.55 ± 105.07	1166.00	117.63 ± 9.12	118.07
EB	10	1284.36 ± 128.71	1279.02	1324.39 ± 134.21	1315.45	103.65 ± 10.50	104.29

Among the three breeds no correlation was found with linear measurements. No significant difference was found with skull index.

## Discussion

Canine skull shape has mainly been evaluated as a criterion for morphology (dolicho/meso/brachycephalic) [19-22]. Specific landmarks are still used for linear measurements [23]. Morphological indices have been applied differently [17,24,25]. Regodon et al. [16], according to criteria proposed by Aguera and Massa [26], showed that both measurements and indices could be obtained radiographically.

Larynx measurements have been conducted in both humans [27,28] and horses [29], but information regarding dogs is sparse [30,31]. Laryngoscopy requires a full knowledge of laryngeal anatomy and function [32,33].

Secondary laryngeal changes can have a significant impact on the overall prognosis of patients with BAOS. These changes have been reported in adult and older dogs as well as in very young patients. For these reasons an accurate evaluation of the larynx could help to justify the poor post-surgical outcome observed in some dogs [2,3,7,34-37].

Considering that actual linear glottic measurements are not influenced by 1st degree laryngeal collapse (everted saccules only), dogs with 2nd and 3rd degree laryngeal collapse were excluded in this study in order not to affect the evaluation. Recently, cephalometric measurements and obstruction in brachycephalic dogs have also been evaluated by Computed Tomographic Imaging [6,38,39].

### Phase I

Glottic linear measurements were adopted instead of glottic area as the transversal diameter (glottic width) is the one that undergoes changes during collapse progression [7]. To measure the transversal diameter, the end phase of expiration was chosen, i.e. when the two arytenoids are at the most adducted position. Besides, the glottic index was adopted as it represents a constant ratio between two actual measurements; if the glottic area is used instead, differences in pixel measurement may arise from different distances between the operator and glottis. As an alternative, a dedicated ruler adjacent to the laryngeal opening would have been valuable in providing a scale [40,41]. In the present study, statistical evaluation of the measurements taken by two different operators demonstrated that results were repeatable.

Direct glottic linear measurements were not significantly different among the three breeds of the study even though a trend for a smaller glottic width was noted in Pugs, followed by FB and EB. Laryngeal height was greater in FB, followed by Pugs and EB. There was a significant difference between the glottic index of EB compared to Pugs, with the latter having a smaller glottic index, i.e. a high and narrow *rima glottis*; FB displayed an intermediate morphology.

### Phase II

Actual skull measurements obtained from radiographs were chosen due to their accuracy compared to direct measurements [16]. The dorso-ventral radiographic view served this purpose, and it was easy to perform and standardize. The correlation between skull length and maximum zygomatic width was adopted in the present study, being considered as indicative measurements of the head morphology [23]. Additionally, laryngeal linear measurements may be easily correlated with linear skull measurements. When skull radiographic measurements were compared to glottic indices, no significant correlation was found. Furthermore, no significant differences regarding skull index were found among the three breeds, indicating similar skull morphology. Therefore, skull index was not considered as being a useful indicator for predicting glottic morphology. On the contrary, it appeared that the glottic index differed among the three breeds, ultimately suggesting to act as a breed-related morphologic indicator of larynx shape (more rounded in EB, more elliptical in Pugs and in-between in FB). The results of this study provide relevant information to clinicians assessing laryngeal anatomy in different brachycephalic breeds prior to surgery. The glottic index may represent an objective way of assessing laryngeal anatomy and monitoring disease progression in different brachycephalic breeds.

Standard surgical treatment for BAOS includes nostril plasty and partial staphylectomy, with saccule resection being more recently questioned [42-44]. Results of surgical correction are often inconsistent, even when performed by skilled surgeons [14,15,42,45,46]. Novel surgical procedures have been recently proposed [47-49] but, regardless of the adopted procedure, some dogs continue to display some residual respiratory noises and exercise intolerance or they can progressively deteriorate. One of the most life-threatening consequence of BAOS is secondary laryngeal collapse [13,50,51] arising from the high negative pressure generated on inspiration. After an advanced laryngeal collapse is established, further treatment is challenging. Recently, a unilateral crycoarytenoid lateralization combined with thyroarytenoid caudo-lateral lateralization (arytenoid laryngoplasty) has been proposed for grade II and III laryngeal collapse, showing promising results in a small cohort of dogs [34]. A particular risk for laryngeal collapse has been previously suggested in Pugs by Torrez and Hunt (3) which are the patients with the lowest glottic index in our study. Further studies are needed to investigate whether a low glottic index may predispose brachycephalic dogs to the development of laryngeal collapse.

## Conclusions

No correlation was found between glottis and skull indices. The lack of correlation between skull and glottic indices does not support skull morphology as predictor of glottic morphology. On the contrary, the glottic index was significantly smaller (high and narrow glottis) in Pugs than in English Bulldogs. The finding of a significant difference on glottis index suggests that the latter may represent, among the three canine breeds considered in the present study, a breed-related morphologic indicator of larynx shape. As a consequence of the lowest glottic index of Pugs when compared to EB and FB, it may be speculated that Pugs’ original narrow glottic width may predispose to further respiratory deterioration possibly as a consequence of progression to laryngeal collapse. However, this hypothesis needs to be validated by further studies.

## Abbreviations

BAOS: Brachycephalic airway obstructive syndrome; EB: English bulldogs; FB: French bulldogs.

## Competing interests

The authors declare that they have no competing interests.

## Authors’ contributions

SR, PB and RC conceived the study, contributed to the acquisition of data and made substantial contributions to conception and design. GL performed the statistical analysis and all authors contributed to interpretation of data, drafting and revision of the manuscript. MC revised critically the manuscript for important intellectual content. All authors read and approved the final manuscript.

## References

[B1] Harvey CE Inherited and congenital airway conditions J Small Anim Pract 1989 30 184 187 10.1111/j.1748-5827.1989.tb01531.x

[B2] Lorinson D Bright RM White RAS Brachycephalic airway obstruction syndrome–a review of 118 cases Canine Pract 1997 22 18 21

[B3] Torrez CV Hunt GB Results of surgical correction of abnormalities associated with brachycephalic airway obstruction syndrome in dogs in Australia J Small Anim Pract 2006 47 150 154 10.1111/j.1748-5827.2006.00059.x 16512847

[B4] Ginn JA Kumar MS McKiernan BC Powers BE Nasopharyngeal turbinates in brachycephalic dogs and cats J Am Anim Hosp Assoc 2008 44 243 249 18762560 10.5326/0440243

[B5] Bernaerts F Talavera J Leemans J Hamaide A Claeys S Kirschvink N Clercx C Description of original endoscopic findings and respiratory functional assessment using barometric whole-body plethysmography in dogs suffering from brachycepalic airway obstruction syndrome Vet J 2010 183 95 102 10.1016/j.tvjl.2008.09.009 18952471

[B6] Grand JGR Bureau S Structural characteristics of the soft palate and meatus nasopharyngeus in brachycephalic and non-brachycephalic dogs analysed by CT J Small Anim Pract 2011 52 232 239 10.1111/j.1748-5827.2011.01047.x 21539567

[B7] Leonard HC Collapse of the larynx and adjacent structures in the dog J Am Vet Med Assoc 1960 137 360 363 14415784

[B8] Coyne BE Fingland RB Hypoplasia of the trachea in dogs: 103 cases (1974–1990) J Am Vet Med Assoc 1992 201 768 772 1399783

[B9] Fasanella FJ Shivley J Wardlaw JL Givaruangsawat S Brachycephalic airway obstructive syndrome in dogs: 90 cases (1991–2008) J Am Vet Med Assoc 2010 237 1048 1051 10.2460/javma.237.9.1048 21034343

[B10] De Lorenzi D Bertoncello D Drigo M Bronchial abnormalities found in a consecutive series of 40 brachycephalic dogs J Am Vet Med Assoc 2009 235 835 840 10.2460/javma.235.7.835 19793013

[B11] Lecoindre P Richard S Digestive disorders associated with the chronic obstructive respiratory syndrome of brachycephalic dogs: 30 cases (1999–2001) Revue Med Vet 2004 155 141 146

[B12] Poncet CM Dupré GP Freiche VG Estrada MM Poubanne YA Bouvy BM Prevalence of gastrointestinal tract lesions in 73 brachycephalic dogs with upper respiratory syndrome J Small Anim Pract 2005 46 273 279 10.1111/j.1748-5827.2005.tb00320.x 15971897

[B13] Wykes PM Brachycephalic airway obstructive syndrome Probl Vet Med 1991 3 188 197 1802247

[B14] Hedlund CS Fossum TW Surgery of the upper respiratory system Small Animal Surgery 2007 3 St. Louis: Mosby Elsevier 817 866

[B15] Harvey CE Upper airway obstruction surgery. II. Soft palate resection in brachycephalic dogs J Am Animal Hosp Assoc 1982 18 538 544

[B16] Regodon S Robina A Franco A Vivo JM Lignereux Y Détermination radiologique et statistique des types morphologiques craniens chez le chien: dolichocéphalie, mésocephalie et brachycéphalie Anat Histol Embryol 1991 20 129 138 10.1111/j.1439-0264.1991.tb00752.x 1897732

[B17] Evans HE Evans HE Axial skeleton, the skull Miller’s Anatomy of the dog 1993 Philadelphia: Saunders 128 133

[B18] Petrie A Watson P Petrie A, Watson P Diagnostic test Statistic for Veterinary and Animal Science 1999 London: Blackwell Science Ltd 168 181

[B19] Onar V A morphometric study on the skull of the German Shepherd dog (Alsatian) Anat Histol Embryol 1999 28 253 256 10.1046/j.1439-0264.1999.00202.x 10488631

[B20] Alpak H Mutus R Onar V Correlation analysis of the skull and long bone measurements of the dog Ann Anat 2004 186 323 330 10.1016/S0940-9602(04)80050-5 15481839

[B21] Fox MW Developmental abnormalities of the canine skull Can J Comp Med Vet Sci 1963 27 219 222 17649461 PMC1583693

[B22] Koch DA Arnold S Hubler M Montavon PM Brachycephalic syndrome in dogs Compend Contin Educ Pract Vet 2003 25 48 55

[B23] Stockard CR The genetic and endocrine basis for differences in form and behaviour as elucidated by studies of contrasted pure-line dog breeds and their hybrids Am Anat Mem 1941 19 1 753

[B24] Brehm H Loeffler K Komeyli H The shapes of the canine skull Anat Histol Embryol 1985 14 324 331 10.1111/j.1439-0264.1985.tb00828.x 2936276

[B25] Nickel R Schummer A Seiferle E Nickel R, Schummer A, Seiferle E Apparato locomotore Trattato di Anatomia degli Animali Domestici 1991 Milano: Casa Editrice Ambrosiana 11 229

[B26] Aguera E Massa R Introducciòn a la topografia craneo-encefalica en el perro basada en metodos radiograficos Arch Zootec 1977 26 9 21

[B27] Eckel HE Sittel C Morphometry of the larynx in horizontal sections Am J Otolaryngol 1995 16 40 48 10.1016/0196-0709(95)90008-X 7717472

[B28] Dailey SH Kobler JB Hillman RE Tangrom K Thananart E Mauri M Zeitels SM Endoscopic measurement of vocal fold movement during adduction and abduction Laryngoscope 2005 115 178 183 10.1097/01.mlg.0000150701.46377.df 15630390

[B29] Lafortuna C Albertini M Ferrucci F Zucca E Braghieri M Clement MG Saibene F Laryngeal movements during the respiratory cycle measured with an endoscopic imaging technique in the conscious horse at rest Exp Physiol 1999 84 739 746 10.1017/S0958067099018515 10481230

[B30] Nasri S Sercarz JA Berke GS Non invasive measurement of travelling wave velocity in the canine larynx Ann Otol Rhinol Laryngol 1994 103 758 766 7944166 10.1177/000348949410301003

[B31] Romussi S Stefanello D Franchini D Cuciti D SICV Aritenoidectomia transorale modificata nel trattamento del collasso laringeo di secondo grado nel cane: valutazioni preliminari Proceedings of the VIII Congresso Nazionale della Società Italiana di Chirurgia Veterinaria: 20–23 June 2001; Olbia 2001 143 149

[B32] Padrid PA McKiernan BC Tams TR Endoscopy of the upper respiratory tract of the dog and cat Small Animal Endoscopy 1999 2 St. Louis: Mosby 357 376

[B33] Holt DE King LG Laryngoscopy and Pharyngoscopy Textbook of Respiratory Disease in Dogs and Cats 2004 St. Louis: Elsevier 109 112

[B34] White RN Surgical management of laryngeal collapse associated with brachycephalic airway obstruction syndrome in dogs J Small Anim Pract 2012 53 44 50 10.1111/j.1748-5827.2011.01156.x 22122300

[B35] Aron DN Crowe DT Upper airway obstruction. General principles and selected conditions in the dog and cat Vet Clin North Am Small Anim Pract 1985 15 891 917 2416110 10.1016/s0195-5616(85)50101-1

[B36] Pink JJ Doyle RS Hughes JML Tobin E Bellenger CR Laryngeal collapse in seven brachycephalic puppies J Small Anim Pract 2006 47 131 135 10.1111/j.1748-5827.2006.00056.x 16512844

[B37] Harvey CE Review of results of airway obstruction surgery in the dog J Small Anim Pract 1983 24 555 559 10.1111/j.1748-5827.1983.tb00400.x

[B38] Schmidt MJ Neumann AC Amort KH Failing K Kramer M Cephalometric measurements and determination of general skull type of cavalier king charles spaniels Vet Radiol Ultrasound 2011 4 436 440 21521397 10.1111/j.1740-8261.2011.01825.x

[B39] Stadler K Hartman S Matheson J O’Brien R Computed tomographic imaging of dogs with primary laryngeal or tracheal airway obstruction Vet Radiol Ultrasound 2011 4 377 384 21447037 10.1111/j.1740-8261.2011.01816.x

[B40] Miller CJ McKiernan BC Pace J Fettman MJ The effects of doxapram hydrochloride (dopram-v) on laryngeal function in healthy dogs J Vet Intern Med 2002 16 524 528 10.1111/j.1939-1676.2002.tb02381.x 12322700

[B41] Demetriou JL Kirby BM The effect of two modifications of unilateral arytenoid lateralization on rima glottidis area in dogs Vet Surg 2003 32 62 68 10.1053/jvet.2003.50000 12520491

[B42] Riecks TW Birchard SJ Stephens JA Surgical correction of brachycephalic syndrome in dogs: 62 cases (1991–2004) J Am Vet Med Assoc 2007 230 1324 1328 10.2460/javma.230.9.1324 17472557

[B43] Poncet CM Dupré GP Freiche VG Bouvy BM Long-term results of upper respiratory syndrome surgery and gastrointestinal tract medical treatment in 51 brachycephalic dogs J Small Anim Pract 2006 47 137 142 10.1111/j.1748-5827.2006.00057.x 16512845

[B44] Cantatore M Gobbetti M Romussi S Brambilla G Giudice C Grieco V Stefanello D Medium term endoscopic assessment of the surgical outcome following laryngeal saccule resection in brachycephalic dogs Vet Rec 2012 170 518 10.1136/vr.100289 22472536

[B45] Harvey CE Upper airway obstruction surgery. I. Stenotic nares surgery in brachycephalic dogs J Am Animal Hosp Assoc 1982 18 535 537

[B46] Harvey CE Upper airway obstruction surgery. III. Everted laryngeal saccule surgery in brachycephalic dogs J Am Animal Hosp Assoc 1982 18 545 547

[B47] Findji L Dupré GP Folded flap palatoplasty for treatment of elongated soft palates in 55 dogs Vet Med Austria/Wien Tierärztl Mschr 2008 95 56 63

[B48] Oechtering GU Hueber JP Noeller C Tremaine H, Wilderjans H, Ness M A multi-level, multi-modal approach to severe brachycephalic airway syndrome Proceedings of the 18th Annual Scientific Meeting of the European College Veterinary Surgery: 2–4 July 2009; Nantes 2009 Zurich: European College of Veterinary Surgeons 478 481

[B49] Dunié-Mérigot A Bouvy B Poncet C Comparative use of CO_2_ laser, diode laser and monopolar electrocautery for resection of the soft palate in dogs with brachycephalic airway obstructive syndrome Vet Rec 2010 167 700 704 10.1136/vr.c5107 21257486

[B50] Venker-van Haagen AJ Venker-van Haagen AJ The larynx Ear, nose, throat, and trachebronchial diseases in dogs and cats 2005 Hannover: Schlutersche 121 165

[B51] Robinson NE Airway physiology Vet Clin North Am Small Anim Pract 1992 22 1043 1064 1523780 10.1016/s0195-5616(92)50300-x

